# The Hidden Role of Non-Canonical Amyloid β Isoforms in Alzheimer’s Disease

**DOI:** 10.3390/cells11213421

**Published:** 2022-10-29

**Authors:** Lukas Busch, Simone Eggert, Kristina Endres, Bernd Bufe

**Affiliations:** 1 Department of Informatics and Microsystems Technology, University of Applied Sciences Kaiserslautern, D-66482 Zweibruecken, Germany; 2 Department of Neurogenetics, Max Planck Institute for Multidisciplinary Sciences, D-37075 Goettingen, Germany; 3 Department of Psychiatry and Psychotherapy, University Medical Centre of the Johannes Gutenberg University, D-55131 Mainz, Germany

**Keywords:** Amyloid Beta, Alzheimer’s Disease, APP, neuroinflammation, glia, microglia, neurodegeneration, cell-surface receptors

## Abstract

Recent advances have placed the pro-inflammatory activity of amyloid β (Aβ) on microglia cells as the focus of research on Alzheimer’s Disease (AD). Researchers are confronted with an astonishing spectrum of over 100 different Aβ variants with variable length and chemical modifications. With the exception of Aβ_1-42_ and Aβ_1-40_, the biological significance of most peptides for AD is as yet insufficiently understood. We therefore aim to provide a comprehensive overview of the contributions of these neglected Aβ variants to microglia activation. First, the impact of Aβ receptors, signaling cascades, scavenger mechanisms, and genetic variations on the physiological responses towards various Aβ species is described. Furthermore, we discuss the importance of different types of amyloid precursor protein processing for the generation of these Aβ variants in microglia, astrocytes, oligodendrocytes, and neurons, and highlight how alterations in secondary structures and oligomerization affect Aβ neurotoxicity. In sum, the data indicate that gene polymorphisms in Aβ-driven signaling pathways in combination with the production and activity of different Aβ variants might be crucial factors for the initiation and progression of different forms of AD. A deeper assessment of their interplay with glial cells may pave the way towards novel therapeutic strategies for individualized medicine.

## 1. Introduction

The neuroinflammatory activity of microglia has, since its discovery, been suspected to contribute to Alzheimer’s Disease (AD) [1,2,3]. Early reports referred to an invasion of reactive microglia at the site of senile plaques [4,5]. Moreover, it was observed that both microglia and astrocytes display exacerbated pro-inflammatory activity in disease-affected brain areas [6]. This led to the theory that glial cells are either directly involved in the pathogenesis of AD or are the immune system’s defensive shield against it [1]. Recent advances have solidified both presumptions and thus placed the inflammatory activity of glial cells as the focus of AD research [3].

**Figure 1:**
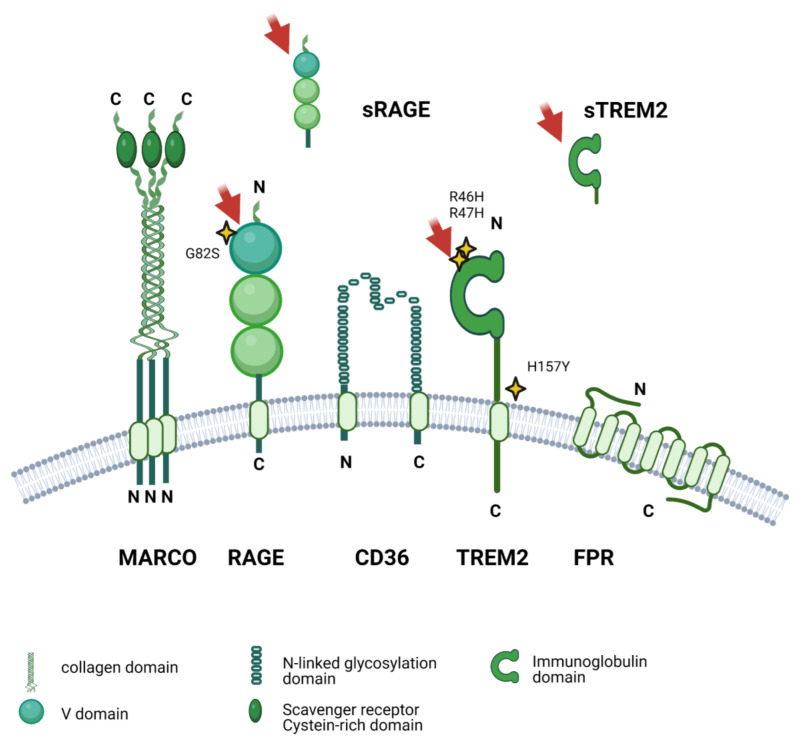
**Membrane topology and domain architecture of microglial Aβ-binding receptors**. Some receptors are shown exemplarily to underpin the diverging domain architecture und potential Aβ-binding site positions as far as identified (red arrows). Relative location of polymorphisms to binding sites are indicated by yellow diamonds. Proportion of domains has been neglected. For RAGE, binding of Aβ is assumed to occur as a dimer on the dimeric receptor [16]. In addition to cleaved soluble RAGE and TREM2, additional soluble splice forms exist [17,18].

AD is closely linked to the deposition of misfolded amyloid peptides in plaques and the occurrence of neurofibrillary tangles consisting of hyperphosphorylated tau protein [7]. While these discoveries were made more than three decades ago [8], the precise role of Aβ in neuroinflammation and neurodegeneration is still incompletely understood [9]. Progress is hampered by some enormous challenges in Aβ research, ranging from a sophisticated peptide process involving multiple proteases [10,11], to complex physiochemical properties of the resulting peptides [12], leading to different oligomeric structures [13] that ultimately elicit miscellaneous physiological effects on neurons, glia, and immune cells [3,7]. We now know that glial cells express a wide array of receptors such as triggering receptor expressed on myeloid cells 2 (TREM2), toll-like receptors (TLRs), and formyl peptide receptors (FPRs), which are capable of directly interacting with extracellular Aβ (see Figure 1). Several other molecules, such as NOD-, LRR- and pyrin domain-containing protein 3 (NLRP3), transient receptor potential cation channel, subfamily M, member 2 (TRPM2), and cluster of differentiation 36 (CD36) are further key modulators of the inflammatory Aβ cascades [14]. More than 100 Aβ peptides with varying lengths, structure, and chemical modifications have been described [10,11,15]. However, most studies focused only on the two most common variants, Aβ_1-42_ and Aβ_1-40_. Thus, the effects of many other fragments that are generated under physiological conditions and are present in significant amounts in affected brain areas remain to be elucidated. We here aim to provide an overview of the wide range of interactions between glial cells and these neglected Aβ variants in the inflammatory cycle of AD. In this context, we discuss the influence of different glia cell types and their cell-surface receptors on APP processing, extracellular cleavage, and chemical modifications of different Aβ species. Finally, we discuss the impact of these variants on neuroinflammation and summarize the current knowledge of gene variants in Aβ receptors on the perception of different Aβ species.

## 2. The Role of Glia in Neuroinflammation

Today we know that an aberrant behavior of both microglia and astrocytes occurs even at early stages of AD where typical cognitive symptoms and neuronal decay are not yet observed [19,20,21].

**Microglia** are the driving force behind the immune defense of the central nervous system (CNS) [3]. As highly motile cells, they survey their environment for pathological stimuli—either from exogenous sources such as bacteria and viruses, or from endogenous structures such as debris of damaged cells and misfolded proteins [22]. Due to its complex structure, the CNS and especially its neuronal network is highly vulnerable to pro-inflammatory stress and cell damage [23]. Therefore, the inflammatory response of microglia has to strike a finely tuned balance between an effective removal of the pathogens and the remaining viability of the surrounding neuronal cells [1,23]. This requires a strictly regulated network of cell-surface receptors and intracellular signaling molecules that carefully control secretion of pro- and anti-inflammatory cytokines and chemokines, generation of oxidative stress through the release of reactive oxygen species (ROS), and phagocytic uptake and degradation of pathogens and cell debris [24]. In addition to these functions, microglia also contribute to the maintenance of healthy brain homeostasis [24,25]. Here, they support neuronal survival and proliferation through constant cross talk with neighboring neurons and astrocytes, and release of trophic factors such as insulin growth factor 1 (IGF-1) or transforming growth factor β (TGFβ) [26,27]. Microglia also help to coordinate the functional state of neurons [28] because they participate in the development of neuronal networks by removing dysfunctional synapses through synaptic pruning [29] and by promoting formation of new synapses through the release of factors such as brain-derived neurotrophic factor (BDNF) [30,31]. Because of their multifaceted roles, microglia have an enormous capability to rapidly change their expression profile and phenotype depending on the encountered stimuli [32]. In a healthy environment, they possess a ramified morphology with wide soma and long branches that help to sense their local environment in order to pursue supportive and vigilant roles. After detection of harmful stimuli, they undergo significant molecular and morphological changes to assume a reactive phenotype with an amoeboid shape and initialize pro-inflammatory processes that help to eliminate pathogens from the CNS [3]. Historically, the amoeboid phenotype has been classified as the pro-inflammatory M1-type and the ramified morphology as the anti-inflammatory M2-type [3]. Recent studies demonstrated that these classifications are an oversimplification because microglia can adopt several different pro- and anti-inflammatory states with distinct gene expression profiles that depend on their local microenvironment and ageing of individual cells [32,33]. In addition, RNA-seq analysis of transgenic AD mice provided evidence for the existence of multiple Disease-Associated Microglia (DAM) subsets, showing unique expression profiles [34] in different forms of neuroinflammation [34,35]. For the sake of clarity, we still refer to the sum of all pro-inflammatory microglial cell types as “reactive microglia”.

Clear evidence indicates that reactive microglia can detect and respond to Aβ. Over the last decade it has become apparent that there is no single interaction partner for extracellular Aβ. Instead, reactive microglia display a large number of different receptors that are potential binding partners. Depending on the environment, disease type, and age of the respective human host, different subsets of reactive microglia exist that express discrete combinations of these receptors that modulate their inflammatory responses. The currently known direct Aβ interaction partners expressed in microglia include structurally diverse molecules such as triggering receptor expressed on myeloid cells 2 (**TREM2**), its co-receptor sialic acid binding Ig-like lectin 3 (**CD33**), cluster of differentiation 36 (**CD36**), toll-like receptors (**TLRs**), formyl peptide receptors **(FPRs**), receptor for advanced glycation endproducts (**RAGE**), chemokine-like receptor 1 (**CMKLR1**), macrophage receptor with collagenous structure (**MARCO**), **Nucleolin**, transient receptor potential cation channel, subfamily M, member 2 (**TRPM2**), and fragment crystallization receptors (**FcRs**) (see Figure 1).

**Astrocytes** are the most abundant cell type in the CNS [36]. They build a supportive framework for neuronal networks that helps to mediate blood flow in cerebral tissues and to regulate the allocation of metabolites and neurotransmitters [36,37]. Astrocytes maintain the integrity of the blood–brain barrier and mediate bidirectional transport processes between the CNS and the periphery [38,39]. Their gate-keeping function also comprises the transport of waste products out of the CNS via the glymphatic system and through the blood–brain barrier (BBB) [40,41]. In addition, astrocytes form a complex communication network that exchanges information through the release of signaling molecules [42]. They are in constant cross-talk with neurons, microglia, and oligodendrocytes to uphold a healthy environment in the CNS, and actively participate in neuronal signal transduction and the control of synaptic plasticity [42]. Similar to microglia, astrocytes are able to undergo gliosis to adopt different reactive states with distinct morphology and gene expression profiles [43]. In a pro-inflammatory environment, astrocytes assume their reactive phenotypes where they suspend many supportive functions. Their reactive forms enable them to compensate microglial dysfunction and to engage in phagocytic roles [44]. Accordingly, astrocytes express many pro-inflammatory modulators that are also found on microglia, including Aβ-interaction partners such as TLRs [45], FPRs, and RAGE [46]. Their interaction with Aβ induces severe functional disturbances such as decreased release of neurotrophic and protective factors and a dysregulation of calcium levels, which in turn leads to massive disruptions in gliotransmission and the induction of apoptotic pathways [47]. In addition, astrocytes are able to internalize Aβ and express a number of Aβ-degrading enzymes such as metalloendoproteases like neprilysin (NEP) and insulin-degrading enzyme (IDE) [48], and the matrix metalloproteases MMP-2 and MMP-9 [49], which helps them to support microglia in the clearance of Aβ (reviewed in [50]). Furthermore, several studies have proposed that astrocytes influence Aβ clearance by mediating the transport of Aβ into the CSF and through the BBB into the bloodstream [51,52,53].

**Oligodendrocytes** are myelinating glial cells of the central nervous system that produce the insulating sheath of axons, which is essential for enhancing axonal action potential conduction [54,55]. In addition, they also contribute to axoglial metabolic support and elimination of oxidative radicals [55]. Not much is known about the involvement of oligodendrocytes in the pathology of AD. However, they express some of the cell-surface receptors that can interact with Aβ such as TLR4 [56,57] and RAGE [58]. Interestingly, recent studies observed molecular heterogeneity of oligodendrocytes in AD mouse models and patients [59,60,61,62,63,64] and three distinct activation states of oligodendrocytes have been identified via single-cell RNA sequencing [65]. Moreover, white matter degeneration and myelin loss have been documented in brains of AD patients [66,67] and studies first theorized that AD might be in part a response to age-related myelin breakdown [68]. Importantly, defects of myelin integrity or demyelinating injuries were recently shown to be drivers of amyloid deposition in vivo [69]. Further detailed investigations are necessary to examine the impact of oligodendrocytes in AD and the contribution of white matter degeneration to AD.

## 3. Processes That Lead to the Generation of Non-Canonical Aβ Variants

**Figure 2:**
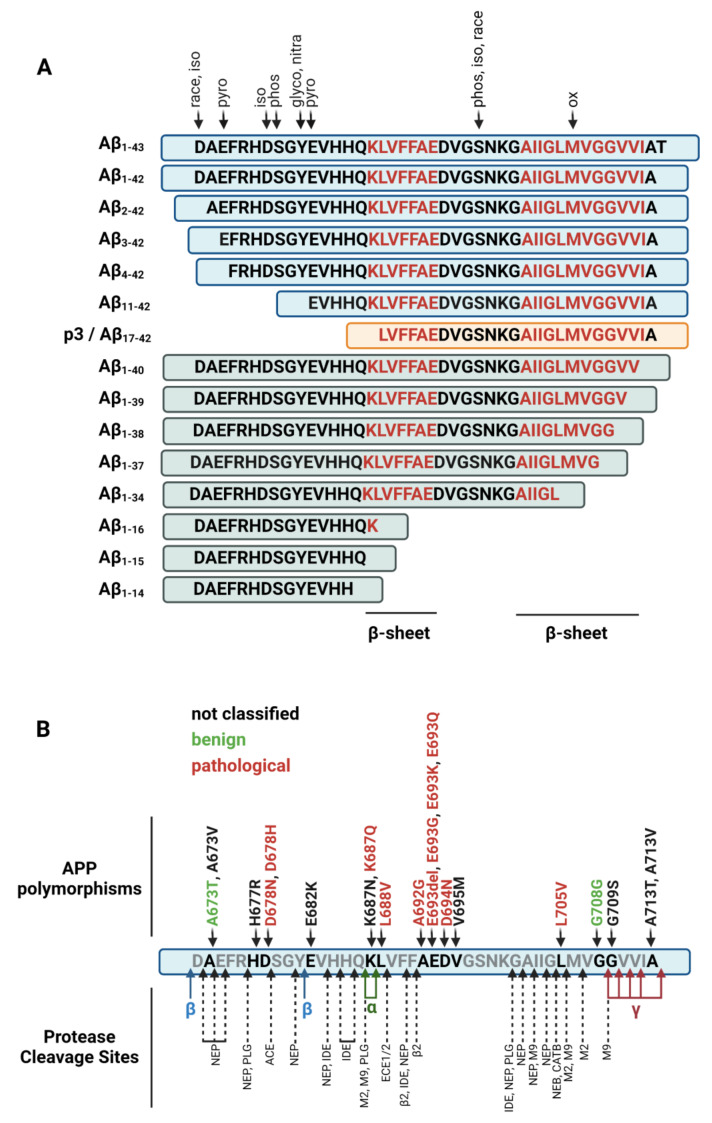
**Truncated and modified Aβ species**. (**A**): Depiction of N- and C-truncated Aβ variants and their potential modification sites as described in Kummer and Heneka, 2014. Motifs for β-sheet formation are indicated in red (race = racemization, iso = isomerization, pyro = pyroglutamylation, phos = phosphorylation, glycol = glycosylation, nitra = nitration, ox = oxidation). (**B**): Aβ peptide with polymorphisms of APP that directly influence the amino acid composition of its Aβ domain (up) [86] and the proposed cleavage sites of various secretases and enzymes (down) [11]. Polymorphisms are classified according to their proposed pathological influence [86]. NEP = neprilysin, PLG = plasmin, ACE = angiotensin converting Enzyme, IDE = insulin-degrading enzyme, M2 = MMP-2, M9 = MMP-9, ECE1/2 = endothelin converting enzyme 1/2, β2 = BACE2, CATB = cathepsin B.

Aβ_1-42_ and Aβ_1-40_ are by far the best-studied variants in AD research but proteolytic processing of APP generates many more fragments (see Table 1). Thus, in a given biological setting, the different glial cell types and neurons are usually confronted with a much wider spectrum of Aβ variants whose properties and cellular effects are yet insufficiently examined. In general, one can distinguish between four different groups of non-canonical variants: N- and C-terminal abridged peptides, elongated peptides, and peptides containing post-translational modifications (PTMs) (see Figure 2A). A given peptide precursor may be subjected to different cleavage forms and modifications, which gives rise to a wide range of possible combinations of truncations and chemical modification (proteolytic conversion of APP and its modification processes are extensively reviewed in [11]). In addition, different Aβ species tend to form heteromeric aggregates with varying structural properties that may trigger further chemical modifications [12,13]. Several lines of evidence argue that these non-canonical Aβ peptides are of importance in the pathogenesis of AD. Firstly, N-abridged species are the most common form of Aβ peptides and comprise about 70% of all Aβ variants found in human brains [8,70]. Second, their levels in the CSF are directly associated with the onset of AD [71]. Third, some chemical modifications such as pyroglutamylated Aβ, which are dominant in AD but not during normal aging, only occur on abridged peptides [72]. Fourth, some familial APP mutations such as the well-known Swedish, French, or German variants alter APP processing and the ratio of abridged Aβ variants, which may directly influence the progression of AD (see Figure 2B) [73].

**Table 1:** **Overview of abridged and/or modified Aβ-species detected in the brain or CSF of AD patients.** Superscript description indicates modification at the respective amino acid (glyco-Y10 = glycosylation at tyrosine at position 10 of the Aβ-domain sequence, ox-M35 = oxidation at methionine at position 35, pyro-E3/11 = pyroglutamylation at glutamate at position 5 or 11, race-D-S26 = racemization of D-conformation serine at position 26).

Aβ Fragments	Modification	Source	References
C-ABRIDGED			
1–13 to 1–20	-	CSF	[74,75]
1–15 to 1–20 ^glyco-Y10^	glyco-Y10	CSF	[76]
1–16 to 1–17	-	Brain, CSF	[74,75,77]
1–20	-	Brain	[70]
1–28	-	CSF	[74]
1–30	-	CSF	[74,75]
1–31	-	Brain	[70]
1–33 to 1–34	-	CSF	[74,75]
1–37 to 1–40	-	Brain, CSF	[74,75,78]
1–37 ^ox-M35^ to 1–40^ox-M35^	ox-M35	CSF	[74]
**N-ABRIDGED**			
2–42 to 11–42	-	Brain	[78,79]
2–40	-	Brain	[79,80]
3–40/42^pyro-E3^	pyro-E3	Brain	[78,80]
4–42 ^ox-M35^ to 5–42 ^ox-M35^	ox-M35	Brain	[70]
4–40	-	Brain, CSF	[74,78]
4–43	-	Brain	[78]
5–40	-	Brain	[79,80]
8–42 ^ox-M35^	ox-M35	Brain	[70]
9–40	-	Brain	[78]
11–42	-	Brain	[80]
11–42 ^ox-M35^	ox-M35	Brain	[70]
11–42 ^pyro-E11^	pyro-E11	Brain	[78,79]
11–42 ^pyro-E11, ox-M35^	pyro-E11, ox-M35	Brain	[70]
17–42	-	Brain	[81]
**C- & N-TRUNCATED**			
2–14	-	CSF	[75]
2–16	-	Brain	[77]
3–15 to 3–17	-	Brain	[74,77]
3–15 to 4–15 ^glyco-Y10^	glyco-Y10	CSF	[76]
3–19 ^pyro-E3^ to 3–20 ^pyro-E3^	pyro-E3	Brain	[80]
3–24 ^pyro-E3^	pyro-E3	Brain	[80]
4–16 to 5–16	-	Brain	[77]
4–17 to 5–17 ^glyco-Y10^	glyco-Y10	CSF	[76]
4–18 to 4–20	-	Brain	[80]
4–23 to 4 -25	-	Brain	[80]
4–34	-	Brain	[80]
4–37	-	Brain	[80]
4–37 ^ox-M35^ to 4–40 ^ox-M35^	ox-M35	Brain	[80]
5–20	-	Brain	[80]
11–23 ^pyro-E11^ to 11–25 ^pyro-E11^	pyro-E11	Brain	[80]
11–27 ^pyro-E11^	pyro-E11	Brain	[80]
11–30	-	CSF	[74]
11–34	-	Brain	[70]
25–35/40 ^race-D-S26^	race-D-S26	Brain	[82]
**CANONICAL FORMS**			
1–38 to 1–40	-	Brain, CSF	[74,78,79]
1–42	-	Brain, CSF	[74,78,79]
1–43	-	Brain	[83]
1–40/42 ^ox-M35^	ox-M35	Brain	[70]
1–40/42^race-D-S26^	race-D-S26	Brain, CSF	[76,82]
1–40/42 ^race-D-D7^	race-D-D7	Brain	[84,85]

**Figure 3:**
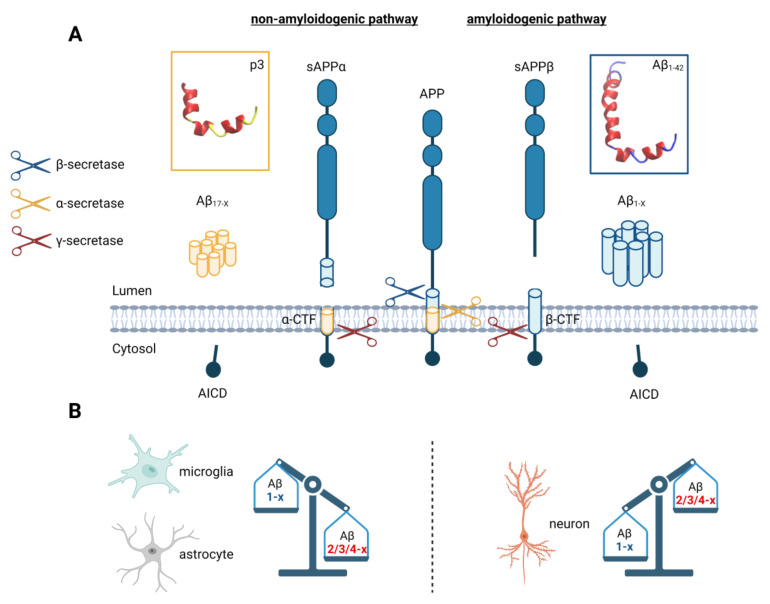
**The amyloidogenic and non-amyloidogenic pathway of APP processing.** (**A**) The non-amyloidogenic pathway is starting by α-secretase cleavage of APP which leads to the release of soluble APPα (sAPPα) and concomitant generation of the α-C-terminal fragment (α-CTF). Subsequent cleavage of the α-CTF by γ-secretase leads to the secretion of the small fragment p3 and release of the APP intracellular C-terminal domain (AICD) into the cytosol. In the amyloidogenic pathway, APP is first cleaved by β-secretase to produce soluble APPβ (sAPPβ) and the membrane retained β-C-terminal fragment (β-CTF). Further processing of the β-CTF by γ-secretase releases AICD as well as the Aβ peptide, which can form neurotoxic oligomers. Aβ production was reported to be higher in neuronal cultures compared to astrocytes or microglia cultures [95,96]. 3D structures (PDB: 1IYT) are based on NMR experiments by Crescenzi and colleagues [97]. (**B**) Glial cells mainly produce N-abridged Aβ peptides (up to 60%) such as Aβ_2/3-X_ or Aβ_4/5-x_, while neurons produce predominantly Aβ peptides starting at position 1 (80%) [98].

Of note, with respect to these non-canonical Aβ variants, data from the currently available animal models are of limited use since the number of non-canonical Aβ variants in these models diverges significantly from those of most human AD patients, and especially the levels of N-abridged peptides are highly reduced [70,87]. This is likely due to the fact that the Aβ sequence from the most common humanized AD mouse models contain specific mutations of the APP gene or genes of its processing enzymes that are found in some rare familiar cases of AD but not in the majority of patients (Figure 2B) [88,89]. For example, the commonly used APP/PS1 mouse line expresses human APP bearing the Swedish mutation K670N/M671L and mutant human Presenilin-1 [90]. Similarly, the popular 5xFAD mouse model expresses human APP with a non-natural combination of the mutations K670N/M671L (Swedish), I716V (Florida), and V717I (London), and human Presenilin-1 bearing the mutations M146L and L286V [91]. Thus, in comparison to the average human population, these mouse models likely have a bias in APP processing [92]. Nonetheless, humans and transgenic mice share two competing principal pathways for APP processing (see Figure 3). Depending on which cleavage process takes place first, this will lead to different N- and C-terminal amyloid variants that can comprise a length of up to 49 residues [93]. An initial cleavage by **α-secretase activity** results in short N-terminally abridged peptides starting at position 17, whereas an initial cleavage by **β-secretases** produces a wide range of Aβ peptides starting at position 1 or 11 [10]. Formally, only peptides that are cleaved at the β-site should be considered as true amyloid β peptides. However, most authors use the “Aβ” abbreviation to also refer to other peptides that are generated through alternative cleavage sites in the amyloid β domain. For simplicity, we will therefore also use this nomenclature, although we are aware that the term amyloid *alpha* would be more appropriate for fragments that are cleaved by **α-secretases**. These enzymes produce the membrane-bound α-C-terminal fragment (αCTF) from APP, which is then processed by γ-secretase into amyloid variants comprising the Aβ-domain 17-X, which are also called **p3 peptides** [94].

**β-secretase** activity leads to the generation of the classical variants Aβ_1-42_ and Aβ_1-40_ mainly via BACE1 (β-site amyloid precursor protein cleaving enzyme 1). BACE1 produces membrane-bound β-C-terminal fragments (β-CTF) by cleaving APP at position 1 (β-site) or at position 11 (β’-site) of the Aβ-domain that are subsequently further processed either by α- or γ-secretases. Cleavage by an α-secretase produces p3 peptides and corresponding N-terminal fragments Aβ_1–14_ to Aβ_1–16_. **γ-secretase activity** is mediated by multiple proteins such as APH-1, PEN-2, Nicastrin, and Presenilin-1 or Presenilin-2 [10]. Peptides that result from **γ-secretase activity** end with residues ranging between position **Aβ _x-37_** to **Aβ _x-43_**. This process is thought to occur through multiple separate intermediate cleavage steps, where mostly three amino acids are cut off within each step [10]. The cleavage process usually starts at position 48 or 49, continues at position 45 or 46, and ends at position 38, 40, or 42. More rarely, peptides ending at position 34, 37, 39, or 43 are generated [10]. The C-terminal variability is due to the imprecise cleavage of γ-secretases whose recognition motifs are not depending on an exact amino acid sequence. In the healthy brain, 50% of the peptides generated this way end at position 40, 16% at position 38, and 10% at position 42 [99]. These ratios shift in AD, leading to a higher production of peptides ending at position 42 [10]. The reason for this is not fully understood; however, mutations of either APP or γ-secretase enzyme complexes can lead to a lowered binding of Aβ to the protease complex, which may then result in a premature release of the cleaved peptides [10]. In addition to these longer variants, γ-secretase can also generate shorter peptides (Aβ_1–14_ to Aβ_1–16_); however, the exact pathways have not been identified, yet [100]. In addition to processing by traditional secretases, Aβ and APP are targeted by a wide range of enzymes and peptidases that can either cut APP directly or interact with Aβ peptides after their generation (see Figure 2B) (reviewed in [11]). These include matrix metalloproteinases such as MMP2 and MMP9, which mainly interact with extracellular or internalized Aβ in glial cells or deposition of fibrillary Aβ aggregates or others such as meprin-β that cleave APP directly in its membrane bound form [11]. This leads to an extensive repertoire of possible peptides (see Table 1).

### 3.1. N-Abridged Aβ Species

**Aβ_2-X_** and **Aβ_3-X_** are thought to be generated by APP cleavage by the metalloprotease meprin-β or after cleavage of “full-length” Aβ_1-X_ by the exopeptidase aminopeptidase A [101]. Not much is known about the differences between Aβ_2-**X**_ and non-abridged variants regarding their biological effects. However, Aβ_2-40_ shows a higher aggregation propensity than Aβ_1-40_ and can seed further aggregate formation of Aβ_1-40_ [102]. Cleavage by meprin-β depends on a specific recognition motif in APP that includes amino acids neighboring the N-terminus of Aβ [102]. This motif is altered in some APP mutants such as the Swedish APP variant, which leads to a diminished processing by these enzymes and a lower number of these variants in patients that carry this mutation [102]. Of note, many popular humanized AD mouse models such as 5xFAD and APP/PS1 mice express the Swedish APP mutation [92]. Thus, these mice have a low amount of Aβ_2-**X**_. By contrast, these N-terminal truncations are frequent in human AD patients [103].

**Aβ_4-X_** variants are abundantly found in dense amyloid plaque cores of human AD patients and 5xFAD mice [104]. Interestingly, Aβ_4-X_ peptides appear to be more commonly released by microglia and astrocytes than by neurons, but the exact processing mechanism is currently unknown [98]. Several studies suggest that a disintegrin and metalloproteinase with thrombospondin motifs (ADAMTS4) and neprilysin may generate these truncations [104,105,106]. ADAMTS4 has been implicated in the generation of variants such as Aβ_4-40_ and Aβ_4-42_ [105]. Both show increased aggregation rates and a similar neurotoxicity as non-abridged Aβ [95,107]. Clearance of oligomeric Aβ_4-40_ and Aβ_4-42_ from the brain is markedly reduced compared to their non-abridged counterparts [108], which may indicate reduced or hampered interactions with glial cells. Of note, injections of Aβ_4-40_ or Aβ_4-42_ into the brain of mice elicit behavioral deficits and impaired memory, which were comparable with those induced by Aβ_1-42_, while injections with Aβ_4-38_ had no effect [95]. Interestingly, transgenic mice expressing human Aβ_4-42_ in their hippocampus do not develop plaques but still suffer from hippocampal pyramidal neuron loss and behavioral deficits [95,109].

**Aβ_11-X_ peptides** are generated by the β-secretase processing pathway after cleavage at the β’-site. Aβ_11-40_ and Aβ_11-42_ levels are commonly elevated in the brain of AD patients [70,79]. They are approximately as abundant as Aβ_1-42_ and can comprise up to 20% of the peptide content in plaques [70,79]. Decreased levels of Aβ_11-X_ in the CSF are associated with the onset of symptoms in AD and have thus been proposed as a biomarker for AD [71]. In plaque cores, Aβ_11-X_ co-localizes with Aβ_1-42_ and is therefore believed to contribute to early plaque formation [110]. In vitro studies demonstrated that Aβ_11-40_ leads to increased neurotoxicity exceeding that of the canonical Aβ_1-40_ [111]. Furthermore, Aβ_11-40_ is capable of rapidly forming fibrils [112] and can therefore act as a cross-seed for the deposition of other Aβ species in plaques [111]. Interestingly, homogenous aggregation of Aβ_11-40_ decreases its neurotoxicity while heterogeneous aggregates of Aβ_11-40_ and Aβ_1-40_ are even more harmful than monomeric or homogeneous oligomers of Aβ_1-40_ [111]. Surprisingly little is known about the effects of Aβ_11-40_ on glial cells. However, we recently showed that Aβ_11-40_ activates the human glial cell line U87 via FPR1 [113].

**Aβ_17-40_** and **Aβ_17-42_** are p3 peptides that are generated via the α-secretase processing pathway. P3 peptides are found in diffuse plaques of AD patients [114] and microglia of AD patients contain various N-terminally abridged Aβ species [115]. Cell culture experiments indicate that they might be produced in even higher amounts than Aβ_1-42_ and Aβ_1-40_ [116]. Of note, the presence of Aβ_17-X_ in the CSF is positively correlated with cognitive impairment of AD patients [71], which argues for a contribution of these peptides to AD. However, they are difficult to detect because many commonly used Aβ antibodies are not selective for Aβ_1-42_ and cross-react with p3 fragments and other N-terminally abridged variants [117]. In addition, many established protocols for isolating Aβ peptides from brain and CSF samples are not suitable for p3 peptides due to their high hydrophobicity and insolubility, which excluded them from many studies that evaluated Aβ levels [118]. Moreover, since several non-canonical proteases such as IDE, NEP and MMP9 have cleavage motifs within the p3 sequence (Figure 2B), it is likely that additional p3 peptides with highly variable C-termini exist. However, these potential variants have not been studied so far. Historically, Aβ_17-X_ were classified as non-amyloidogenic because they did not display fibrillary aggregation and deposition. However, recent advances have cast doubt on these assumptions because conflicting data regarding the amyloidogenic capabilities of p3 peptides exist (reviewed in [94]). While early reports found no evidence of toxic fibril formation by p3 peptides [119], more recent studies found that p3 peptides form fibrils and soluble oligomers [120,121,122] with even faster aggregation kinetics than Aβ_1-40_ or Aβ_1-42_ [107,120]. Aβ_17-40_ and Aβ_17-42_ induce pro-inflammatory cytokine production in human and murine glial cell lines, leading to neuronal decay through pro-inflammatory caspase-activation in neuronal cell lines [107,123], which triggers pro-inflammatory activity after injection into mouse brains [124]. Moreover, we recently showed that all human FPRs can detect Aβ_17-40_ and that FPR1 is activated at 30-fold lower concentrations by the peptide than by Aβ_1-42_ [113].

### 3.2. C-Terminal Variants

**Aβ_1–43_** is produced by γ-secretase cleavage and can be found abundantly in amyloid plaques [125,126,127]. Its levels are heightened in both spontaneous and familiar AD patients, but not in age-matched controls [128]. In particular, familiar AD cases with mutations in the γ-secretase subunit PS1 show an elevated generation of Aβ_1–43_ compared to other species [129,130]. Unlike shorter variants, only low amounts of Aβ_1–43_ are found in the CSF [131] and cerebral blood vessels [125]. Aβ_1–43_ is highly amyloidogenic and neurotoxic in mouse neuronal primary cultures and cell lines [132,133]. Furthermore, injections of Aβ_1–43_ into APP mice lead to severe depositions of Aβ_1–42_, indicating the potent capability of Aβ_1–43_ to seed the aggregation of other variants [134].

**Aβ_1–37_** to **Aβ_1–39_** are produced by the traditional β-secretase pathway. Compared to canonical Aβ_1–42_ and Aβ_1–40_, these C-abridged peptides appear to be harmless or at least significantly less neurotoxic [135,136]. Aβ_1–38_ forms oligomers and fibrils, but its low abundance in plaques indicates that it is easily cleared from the CNS. Furthermore, levels of these C-abridged peptides in CSF are altered in patients with AD [137]. Functionally, C-abridged Aβ species can act as a scavenger for Aβ_1–42_ and Aβ_1–40_ because they can form aggregates with these peptides, leading to reduced synaptic disruption, reduced neurotoxicity, and lowered amyloidogenicity [136]. In a *Drosophila melanogaster* model, expression of Aβ_1–42_ led to severe neuronal and behavioral pathologies, but co-expression of either Aβ_1–37,_ Aβ_1–38_ or Aβ_1–39_ attenuated these effects [135]. In addition, a lower ratio of soluble Aβ_1–42_/Aβ_1–38_ (i.e., a higher Aβ_1–38_ content) was associated with a later age-at-death in male AD patients [136].

**Aβ_1–34_** is present in the brain and CSF of AD patients and AD mouse models [138,139]. Its levels are highly increased in the CSF of AD patients compared to healthy controls [139]. Interestingly, Aβ_1–34_ is also commonly found in brain vessels in early AD stages but diminishes with further progression of the disease [139]. Aβ_1–34_ generation processes have not been fully elucidated. In cell culture experiments and transgenic mice, generation of Aβ_1–34_ depended on initial BACE1 activity [139], but its C-terminal truncation is likely produced through procession of Aβ_1–40_ or Aβ_1–40_ by glia-derived metalloproteases such as MMP2 and MMP9 [140]. Thus, Aβ_1–34_ is thought to be a degradation marker for clearance activity of microglia in early stages of AD [138,139,140]. Interestingly, an in vitro study reported that, Aβ_1–34_ protected APP-expressing HEK293 cells against caspase-3-mediated apoptosis, which may indicate that Aβ_1–34_ is not just a side product of degradation processes but may also have resolving properties [141].

**Aβ_1–14_** to **Aβ_1–16_** are commonly generated during APP processing but are thought to be harmless [142] because they do not elicit pro-inflammatory glia signaling, are not capable to form amyloidogenic aggregates, and are most likely quickly degraded. Increased levels of these fragments in the CSF indicate increased β-secretase activity and have thus been proposed as a biomarker for Aβ production [78,100].

### 3.3. Post-Translational Aβ Modifications (PTMs)

**Pyroglutamylation** refers to the conversion of glutamate into pyroglutamate, which can occur in N-abridged Aβ peptides at position 3 (Aβ_3-x_^pyro-E3^) and position 11 (Aβ_11-x_^pyroE11^). This process was shown to be catalyzed by glutaminyl cyclase in vitro [143,144] and also in vivo [145,146]. Pyroglutamate highly influences the secondary structure of Aβ, leading to an enhanced β-sheet structure, higher hydrophobicity, and increased aggregation propensity [147]. Aβ^pyroE^ species are peculiarly abundant in senile plaques [78,148] and are also present in diffuse, soluble aggregates [149,150]. Aβ_3-X_^pyro-E3^ is highly amyloidogenic and can seed the aggregation of other Aβ species, which increases fibril formation significantly [151,152]. Such cross-seeded aggregates appear to be more neurotoxic than homogenic aggregates and may thus contribute to the pathological effects of AD [152]. Unlike most Aβ variants, Aβ^pyroE^ is only associated with the progression of AD but not with ageing [72]. In pre-clinical AD patients without senile plaque formation, Aβ^pyro^ is not initially present within soluble aggregates [149] but appears during further progression of the disease when patients experience their first cognitive symptoms [149,153]. Deposition of Aβ_3-40_ ^pyro-E3^ into insoluble plaques precedes deposition of Aβ_1–42_ and Aβ_1–40_ [148] and may thus be involved during the onset of early symptoms. Aβ_3-X_ ^pyro-E3^ species also accumulate in lysosomes of microglia [154] where they are suspected to cause lysosomal failure, microglial decay, and subsequent progression of AD [155,156]. In addition, pyroglutamylation may affect protease-mediated decay, and influence the capabilities of Aβ to interact with cell-surface receptors. For example, murine NMDAR recognizes Aβ but not Aβ_3-X_ ^pyro-E3^ [157]. Furthermore, experiments with porcine primary microglia revealed that Aβ_3–42_^pyro-E3^ is more potent to enhance *E. coli* phagocytosis than the non-abridged peptide (360). Unfortunately, production and deposition of Aβ^pyroE^ in most AD animal models is not comparable with human AD patients because in APP transgenic mouse lines initial plaques are devoid of Aβ_3-X_ ^pyro-E3^ species [158,159,160]. Nonetheless, in some cases mouse models can provide valuable insights into the biological effects of Aβ^pyroE^ species. For example, 5xFAD mice overexpressing the human glutaminyl cyclase show an increased Aβ^pyroE^ production, plaque formation, and behavioral symptoms, while the glutaminyl cyclase knockout reduces plaque deposition and rescues behavioral performance [145]. Moreover, treatment of APP/PS1 mice with antibodies against Aβ_3-X_^pyro-E3^ ameliorates behavioral symptoms and decreases amyloid plaque numbers, which is likely due to FcR-mediated clearance by microglia [161].

**Isomerization** of Aβ occurs mainly at asparagine (N) and aspartate (D) residues and is commonly detected at position 1 (Aβ^iso-D1^) and 7 (Aβ^iso-D7^) [162]. Isomerization happens through spontaneous, non-enzymatic reactions. Thus, its probability increases throughout the lifetime of Aβ [163]. In plaque core preparations, Aβ species with *iso-D1* and *iso-D7* are more common than other variants and are present in higher concentrations than in “younger” diffuse plaques or vascular depositions [164,165]. Of note, isomerization has a strong impact on the secondary structure of Aβ and leads to faster aggregation and deposition [166,167]. Aβ^iso-D7^ also shows an increased neurotoxicity through excessive NO generation [168,169] and inhibits the α7 Nicotinic receptor, which has been implicated in long-term memory formation [168]. Microglia can internalize isomeric Aβ [164]; however, isomeric Aβ is more resistant against degradation in microglial lysozymes [163] and against intracellular and extracellular proteases [163,170], which fosters lysosomal failure [155,156].

**Phosphorylation** can potentially occur at serine residues 8 (Aβ^pho-S8^) and 26 (Aβ^pho-S26^) and at tyrosine residue 10 (Aβ^pho-T10^) [13]. These modifications are thought to occur mainly through extracellular kinases or after internalization. **Aβ^pho-S26^** accumulates in early AD stages in neurons, but only low concentrations are present in extracellular plaques [171,172]. It can assemble into soluble oligomers that do not form fibrils and exhibit increased neurotoxicity in cell culture experiments [172]. **Aβ^pho-S8^** levels are elevated in later stages of AD where it mainly occurs in compact plaques [173]. A phosphorylated serine at position 8 increases the stability of Aβ^pho-S8^ aggregates by attenuating their recognition by degrading enzymes and inhibiting microglial clearance [174,175]. Due to the clearance resistance, Aβ^pho-S8^ species may contribute to plaque spreading [175]. In this context, Hu and colleagues showed that Aβ_1-42_^pho-S8^ can cross-seed with non-modified Aβ_1-42,_ which generates aggregates with elevated neurotoxicity [152].

**Oxidation** is mainly caused by radicals such as ROS and NO and can occur at the methionine (M) at position 35 (Aβ^ox-M35^). Aβ^ox-M35^ molecules are abundantly released by reactive microglia. Oxidation disrupts fibril formation and destabilizes oligomer formation [176,177]. Since generation of oxidative stress is a hallmark of neuroinflammation, the amount of oxidized Aβ is thought to increase during the progression of AD. Oxidized Aβ species with varying length have been identified in brain and CSF samples of AD patients [70,74,80]. A study by Head and colleagues found oxidized species in 46% of diffuse plaques and in 98% of cored plaques [178]. Oxidized Aβ was found in plaque-invading microglia of AD patients [178]. However, so far it has not been elucidated if oxidation influences interactions between Aβ and microglia.

**Racemization** converts amino acids from their L- into D-conformation. For Aβ, racemization was observed in aspartate residues (D) at position 1 (Aβ^D-D1^) and in seryl residues (S) at position 26 (Aβ^D-S26^) [13]. Racemization of aspartic acid increases the fibrillary aggregation kinetic [179]. In addition, further modifications such as sylation and nitration were observed [13]. However, their biological relevance is yet unclear. Some studies suggest that these PTMs may also influence the aggregation properties of Aβ and could influence cross-seeding with other Aβ species [152].

### 3.4. Splice Variants of APP

The APP gene is highly conserved in mammals, consists of 18 exons, and spans over 290 kilobases [89,180]. Ten APP splice variants have been described that range between 639 to 770 amino acids [89]. The three variants **APP695**, **APP751**, and **APP770** make up almost the complete protein quantity [181,182]. APP770 is the longest isoform and contains all exons [181,182]. In comparison, APP751 does not include exon 7, which encodes for an additional Kunitz-type protease inhibitor (KPI) domain that protects the protein against proteolytic cleavage by certain enzymes [183]. APP695 lacks both exons 7 and 8,and is therefore deficient of the KPI domain from exon 7 and an OX-2 domain from exon 8, which is thought to influence cell-surface binding [184]. All three mayor isoforms contain the full Aβ sequence and can thus potentially be processed into Aβ and p3 peptides. Interestingly, APP695 is the most common isoform in the brain and is mostly expressed by neurons, whereas APP751 and APP770 are more dominant in glial cells [181,182,184].

### 3.5. APP Processing Is Different in Neurons, Astrocytes, Microglia, and Oligodendrocytes

**Neurons** are beyond doubt the primary source for the canonical Aβ_1-40_ and Aβ_1-42_ variants [185,186]. They mostly express the APP isoform APP695 and only minor amounts of APP751 and APP770 [187,188]. Neuronal mRNA levels of APP are approximately 10-fold higher [189] than in other cell types and neurons produce approximately four-fold more soluble APP proteins than astrocytes and microglia [190]. In line with this, Aβ production was also shown to be elevated in neuronal cultures compared to astrocytes or microglia cultures [96]. This is corroborated by a study reporting a ~7× higher generation of Aβ_1-40_ secreted by neuronal cultures compared to Aβ released from astrocytic cultures [98]. Neurons predominantly secrete Aβ peptides starting at position 1 (80%), whereas those released from astrocytes and microglia represent mainly N-terminally abridged Aβ peptides including Aβ_2/3-x_ and Aβ_4/5-x_ (Figure 3B) [98], suggesting that secretase levels are different in the respective cell types. In line with this, BACE1 protein levels were reported to be more abundant in cultured neurons compared to astrocytes, whereas BACE2 showed a higher presence in astrocytes [191]. Furthermore, neurons produce 3.4 times more Aβ_17-x_ than Aβ_1-40-43_, while astrocytes generate 7.8 times more Aβ_17-x_ than Aβ_1-40-43_, indicating a higher rate of α-secretase cleavage in astrocytes compared to neurons [96]. Protein expression of PS1, the catalytic domain of the γ-secretase complex, was shown to be comparable in neurons and astrocytes, suggesting no major difference regarding γ-secretase processing in both cell types [191].

**In astrocytes**, all three major splice forms of APP, APP695, APP751, and APP770, were shown on the protein level [192]. Splice forms APP751 and APP770 seem to be predominant [190,192], while APP695, which is the major described APP splice form in neurons [187,188], has a lower abundance. This might contribute to differences regarding the proteolytic conversion of APP between astrocytes and neurons. In contrast to neurons, only 40% of the secreted Aβ peptides are cleaved at position 1 in astrocytes [98]. This is of interest, because N-terminally abridged Aβ species are prevalent in neuritic plaques [77,95,193,194]. N-terminally abridged Aβ species from astrocytes and microglia may therefore contribute to a higher proportion to the formation of neuritic plaques than peptides derived from neurons. In line with this idea, cell culture experiments demonstrated that astrocytes and microglia indeed secrete higher amounts of N-terminally Aβ variants such as Aβ_2/3-40_ and Aβ_4/5-40_ [98]. Proteolytic conversion of these variants does not depend on BACE1 activity but is likely mediated by plasma-membrane associated cathepsin B (CatB) [195]. However, a number of other proteases such as meprin β [196], neprilysin [197], myelin basic protein [198], the metalloproteinase ADAMTS4 [105], and aminopeptidases [199] may also contribute to the production of these N-terminally modified Aβ variants [11,200]. By contrast, the proportions of the C-terminal Aβ variants Aβ_1-37_, Aβ_1-38_, Aβ_1-39_, and Aβ_1-42_ to Aβ_1-40_ did not differ between neurons, astrocytes, and microglia [98], indicating no differences in the C-terminal Aβ peptide-trimming by γ-secretase between these different cell types [10]. Cortical astrocyte cultures have lower BACE1 protein levels and higher levels of BACE2 than corresponding neuron cultures [191]. In line with the finding that APP is processed by BACE2 at position 19–20 and 21–22, Western blot experiments demonstrated a more efficient generation of Aβ_1-15_, Aβ_1-19_, and Aβ_1-20_ in astrocytes of APP/PS1 mice [191]. However, the isolation of primary astrocytes typically yields cultures with mostly reactive phenotypes [201], and these therefore possess presumably higher APP protein levels than resting astrocytes since it has been shown that APP expression in reactive astrocytes is substantially increased following neuronal damage [202]. By contrast, only little APP expression is documented in resting astrocytes in vivo [203]. Therefore, primary cultures may not exactly reflect the in vivo situation of the cell types analyzed. Of note, BACE1 protein and APP levels can be induced by 300 to 600% through stimulation of astrocytes by cytokine combinations or Aβ_1-42_ [204].

In **microglia,** APP protein expression was reported at an early stage [205,206]. All three major splice forms, APP695, APP751, and APP770, are present in microglia cell cultures [192]. However, microglia express more than 50% of their APP mRNA as transcripts as APP695, which encoded for the KPI-domain; approximately 22% of total APP mRNA represented APP770 mRNA, 45% APP751/L-APP752 mRNA, 25% L-APP733 mRNA, and approximately 4% APP695 and L-APP677 [207]. Furthermore, BACE1 expression has been indicated on a protein as well as the mRNA level in microglia cells [208]. The presence of α-secretase ADAM10 in microglia was also shown via an upregulation of ADAM10 after reduction in cortical activity [209]. Interestingly, protein amounts of gamma secretase subunits such as PS1 and Nicastrin are increased in activated microglia [210].

The whole APP processing machinery is present in microglia under certain conditions. However, the impact of microglia on APP processing has not been characterized extensively, yet. Microglia produce mainly N-terminally modified Aβ peptides including Aβ_2/3-x_ and Aβ_4/5-x_ [98], suggesting a possible contribution of N-abridged Aβ species from microglia to the peptides found in vascular and neuritic plaques [77,194]. Several studies with the immortalized mouse microglial cell line BV-2 revealed an interaction of microglia with the extracellular matrix that affects APP secretion as well as the intracellular biogenesis of APP [207]. APP expression and release of soluble APP was highest after adherence to uncoated plastic surfaces [207]. A further study using this cell line showed that Aβ_25-35_ and lipopolysaccharide treatment induced Aβ secretion [211]. APP is expressed on the surface of microglia cells and it is upregulated due to their activation [205,206]. Direct stimulation of APP with agonist antibodies also led to robust activation of microglia [212,213]. Even an activation of microglia via Aβ-binding to APP has been indicated [214]. Collectively, these data indicate that APP itself can serve as a cell-surface receptor that helps regulating microglial activation.

In **oligodendrocytes,** analysis of mRNA transcripts identified the APP splice forms APP695, APP751, and APP770 [215]. Interestingly, a lower molecular weight form of full-length APP was more substantially expressed than higher molecular weight forms, suggesting that oligodendrocytes, similar to neurons [216], may express more APP695 than APP751 and APP770 [217]. Adult oligodendrocyte progenitor cells (aOPCs), which were purified from a >4-month-old rat brain, generally expressed BACE1, APP, and components of the γ-secretase complex such as Presenilin-1, anterior pharynx-defective 1 (APH1), and Nicastrin [218]. APP protein expression in oligodendrocytes has also been shown via immunostaining of rodent brain [219,220,221], as well as in cell culture [215,217,222]. Moreover, ADAM10 expression has been documented in oligodendrocytes of the developing brain at later embryonic stages [223]. APP processing analyses in a cell line of murine OPCs (Oli-neu) [224] documented generation of the APP intracellular domain (AICD) by γ-secretase and production of an APP C-terminal fragment, which increases after treatment with a γ-secretase inhibitor [225]. A more detailed study even addressed various differentiation stages of cultured aOPCs and APP processing [218]. These aOPCs can be maintained for several months in media containing Fibroblast growth factor 2 (FGF2) which promotes survival and proliferation of aOPCs positive for NG2 (chondroitin sulfate proteoglycyan NG2/CSPG4), a marker for aOPCs [218]. Interestingly, FGF2 withdrawal increased expression of full-length APP as well as Aβ_1-42_ production. aOPC cultures including only 0 or 1 ng/mL FGF2 secreted ~four-fold higher ratios of Aβ_x-42_ to total Aβ than cultured fetal rat neurons, suggesting a high rate of γ-secretase processing in these aOPC cultures [218]. α-CTF accumulated in media without FGF2 while β-CTF and the p3 fragment increased with elevated FGF2 concentrations, suggesting that γ-secretase prefers the APP α-CTF as a substrate at higher concentrations of FGF2. Further proteases that are capable of processing Aβ have also been found in oligodendrocytes. One example is ADAMTS4, which is exclusively expressed in oligodendrocytes in mouse brain and generates Aβ_4-x_ from both full-length APP and Aβ_1-x_ [105]. Mass spectrometric profiles of different Aβ species from 5-day-old OPC cultures from WT and ADAMTS4 KO mice confirmed release of Aβ_4-x_ species from these cells [105]. Interestingly, ADAMTS4 mRNA levels were increasing during the culture period of 5 days, suggesting a higher conversion of APP by ADAMTS4 in myelinating oligodendrocytes [105]. Oligodendrocytes therefore seem to be a source of secreted Aβ_4-x_ peptides [105], which corresponds to the finding of N-terminally abridged Aβ variants such as Aβ_2/3-40_ and Aβ_4/5-40_ secreted by microglia and astrocytes in contrast to neurons [98].

## 4. Cellular Responses of Glial Cells towards Aβ

### 4.1. Contribution of Aβ Internalization and Degradation

**Figure 4:**
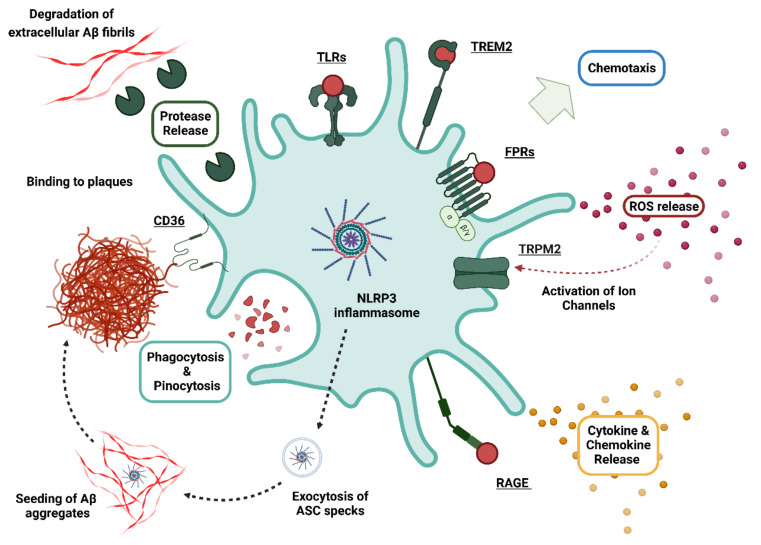
**Cellular responses of microglia after detection of Aβ.** Microglia are capable of perceiving soluble Aβ peptides and insoluble aggregates via multiple cell-surface receptors, which then induce varied cellular responses such induction of cell migration, cytokine and chemokine release, secretion of proteases, and generation of oxidative stress [3,23]. These processes are in part regulated by the intracellular NLRP3 inflammasome. The release of inflammasome components (ASC specks) may lead to seeding of Aβ and may thus contribute to plaque formation [248].

Microglia help to remove toxic peptide Aβ isoforms from the CNS via internalization and subsequent degradation by several mechanisms (see Figure 4) [226]. Various microglial receptors are able to mediate uptake of Aβ by endocytosis via formation of receptor-peptide complexes. However, different aggregates seem to preferentially interact with different types of cell-surface receptors. For example, FPRs help in the clearance of Aβ_1-42_ monomers and small oligomers [227,228,229], whereas fibrillary aggregates are not efficiently internalized [230,231]. Scavenger receptors such as SR-A [232], SR-B1 [233], and CD36 [234,235] appear to prefer the uptake of fibrillary forms of Aβ_1-42_, whereas TLRs have been reported to mediate the internalization of monomeric, oligomeric, and fibrillary Aβ_1-42_ [226]. TLR2 [236,237] and TLR4 [238] induce the uptake of Aβ_1-42_ and trigger internal degradation processes. In addition, microglia can also internalize Aβ_1-42_ via unspecific uptake mechanisms, such as pinocytosis through phosphoinositid-3-kinase (PI3K) [239] or Ras-related C3 botulinum toxin substrate 1 (Rac1)-dependent pathways [240]. Internalization leads to different internal degradation processes in microglia. In cell culture experiments, fibrillar Aβ_1-42_ was effectively degraded through autophagy by lysosomes [241,242,243]. The acidic environment in lysosomes enables the formation of compact Aβ_1-42_ aggregates that are resistant against further degradation [155,156]. Microglia can release these aggregates as microvesicles into the extracellular space where they contribute to plaque formation and neurotoxic effects [155,156]. This can trigger a vicious cycle of apoptotic pathways [155] involving an aberrant activation of the NLRP3 inflammasome [244] and the apoptosis of microglia [155]. Some studies suggest that the release of degradation-resistant Aβ_1-42_ from dying microglia might be the driving force behind plaque deposition since microglia-deficient animal models show only minimal Aβ_1-42_ plaque formation [245,246]. Interestingly, autophagy of Aβ in microglia seems to become less efficient with disease progression [247].

### 4.2. Contribution of NLRP3 Signaling

**Figure 5:**
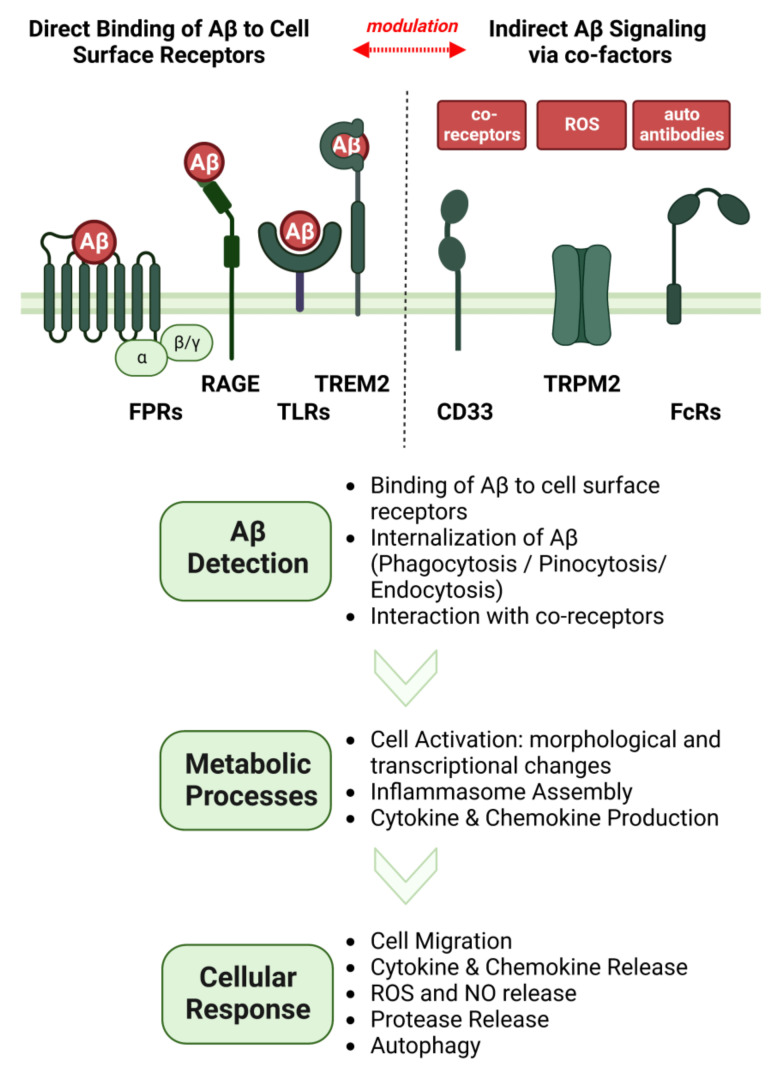
**Aβ-mediated activation of microglia.** Direct detection of Aβ (e.g., by TREM2, TLRs, FPRs, RAGE) or indirect stimulation through Aβ-mediated processes (e.g., TRPM2 activation by ROS, detection of autoantibodies against Aβ by FcRs) leads microglia to adopt their reactive phenotype in which they utilize intracellular signaling pathways and metabolic processes to initiate their pro-inflammatory response.

The wide range of interaction partners for Aβ leads to a number of equally diverse and complex interactions between different signaling cascades in the inflammatory response of microglia (see Figure 4 and Figure 5) [3,23]. These cascades are mainly regulated by inflammasomes, which are cytosolic protein complexes that mediate inflammatory responses of immune cells [249]. In AD, formation of the NLRP3 inflammasome and subsequent caspase-1 activity have been proposed to be driving forces in the microglial response to Aβ [248]. The main components of this inflammasome are the cytosolic receptor NLRP3, the adaptor protein adaptor protein apoptosis-associated speck-like protein containing a caspase activation and recruitment domain (ASC), and the inactive precursor form of caspase-1 (pro-caspase-1) [248,250]. Priming and assembly of the NLRP3 inflammasome is initiated by an activation of NF-κB pathways that are typically induced by extracellular stimulation via surface receptors [248] such as TLRs [236], CD36 [251], and FPRs [252]. This leads to NLRP3 upregulation, which then recruits ASC and pro-caspase-1 for inflammasome formation [3,248]. Upon a second signal, which is either triggered by cell-surface receptors or through direct interaction of NLRP3 with internalized Aβ [253], the primed inflammasome complex autocatalyzes pro-caspase-1 through the adaptor protein ASC into its active state, which subsequently triggers transformation of interleukines and cytokines into their biologically active forms [248]. Of note, microglia release inflammasome components such as ASC specks into the extracellular space, where they can be a seed for Aβ, which may propagate plaque formation [254]. In general, detection of Aβ induces the release of Il1β, IL6, IL8, IL33, IL34, and other inflammatory factors such as TNFα. IL1 and its isoform Il1β belong to the most prominent representatives because upregulation of Il1β already occurs at the earliest stages of plaque evolution [255]. Il1β release increases APP production in neurons [256,257] and influences APP processing via upregulation of proteases [258,259,260]. High IL1 levels were found in brain tissue [261,262] and cerebrospinal fluid [261] of AD patients and APP/PS1 mice [262]. Il1β induces further self-production in microglia and astrocytes, leading to a self-potentiating inflammatory feedback loop [263]. This is associated with neuronal toxicity, exacerbated synaptic loss and aberrant glial activation.

### 4.3. Contribution of Oxidative Stress

Generation of ROS and radical nitrogen species (RNS) is a typical pro-inflammatory action of microglia to remove extracellular pathogens [3,264]. Microglia can release an oxidative burst, which is usually driven by the transmembrane enzyme complex NADPH oxidase (NOX) [264]. NOX activity is triggered as a secondary effect after the phagocytosis of pathogens or the detection of PAMPS or DAMPS by cell-surface receptors such as TREM2 [265] and FPRs [266]. This leads to the generation and release of ROS into the extracellular space. Unfortunately, this does not only target harmful pathogens but also harms neighboring cells, beneficial proteins, and lipids [264]. The healthy brain combats oxidative stress through a complex antioxidant system, which includes radical scavengers such as glutathione that protect cell organelles from damage and dysfunction [264,267]. However, aberrant release of ROS and RNS can overwhelm this antioxidant defense system, leading to severe damage through lipid peroxidation, protein oxidation, and DNA damage [264].

Generation of oxidative stress has been described as a hallmark feature of AD [7]. Signs of oxidative damage are commonly found in the brain, CSF, blood, and urine of patients [268,269,270,271]. In particular, brain areas afflicted with large plaque and NFT depositions show high levels of oxidated proteins and lipid peroxidation. Next, regulatory factors of NOX are upregulated in AD brain tissue [272,273]. Aβ triggers ROS release through activation of microglial cell-surface receptors. The ROS effects are initially dampened by the antioxidant system, but chronic exposure of cells to Aβ leads to its depletion and thus the subsequent exacerbation of oxidative damage [274]. Aβ-mediated oxidative stress then disrupts synaptic signaling, induces astrocyte activation, and leads with increasing levels to neuronal death. In addition, ROS activates TRPM2, which propagates the pro-inflammatory activity of microglia and initiates apoptotic pathways in neurons [275]. Apoptotic cells subsequently release additional DAMPs that are perceived by microglia and further stimulate the pro-inflammatory machinery, thus leading to the self-renewal of oxidative stress. Moreover, oxidative stress leads to increased production of Aβ through modulation of APP procession pathways [276,277,278,279]. Treatment of neuronal cell lines with H_2_O_2_ increases expression and activity of β- and γ-secretases that mediate the production of pathogenic Aβ species [276,278,279]. In particular, γ-secretase activity seems to be susceptible to the effects of oxidative stress and may result in increased β-secretase levels through yet unknown pathways [279,280]. In addition, oxidative stress can directly facilitate the generation of modified Aβ species through oxidation at position 35 or nitrosylation at position 10 [13].

### 4.4. Interaction of Aβ with Other Neuropathological Proteins

**Amylin** (also known as **islet amyloid polypeptide** (IAPP)) is a peptide hormone generated by islet β cells in the pancreas that is co-secreted with insulin into the blood stream during regulation of blood glucose levels [281]. Amylin-Aβ aggregates are present in cerebral plaques of patients with familiar AD [282]. In CSF samples of familiar AD patients, high Amylin concentrations are associated with decreased Aβ_1-42_ levels, which suggests that Amylin may influence Aβ_1-42_ transport between the brain, CSF, and blood [282,283]. Interestingly, the Amylin receptor may also be able to interact with Aβ [284]. Amylin and Aβ_1-42_ share a sequence homology of more than 50% and form similar β-sheet structures. Moreover, Amylin can act as a seed for Aβ_1-42_, which can lead to amorphous heterocomplexes that display a highly increased neurotoxicity compared to either pure Amylin and Aβ_1-42_ aggregates [285].

**Prion protein (PrP)** is a cell-surface protein encoded by the *PRNP* gene, which is ubiquitously expressed throughout human tissues, but is most abundant in the brain (reviewed in [286]). Here, PrP is mainly expressed in astrocytes and neurons, but low levels are also present in microglia and oligodendrocytes [287,288,289]. In addition to its membrane-anchored isoform, soluble PrP variants have been described [290,291]. PrP’s biological functions are not fully elucidated but several studies argue for PrP contribution to synapse formation and neuronal signal transduction [292,293,294]. Its physiological form is dominated by an α-helical secondary structure and is referred to as PrP^c^ (cellular form) [286]. However, under pathological condition, PrP undergoes a structural shift into a neurotoxic isoform with predominant β-sheet conformation, also called PrP^sc^ (scrapie form) [295]. Formation of PrP^sc^ is thought to be contagious, leading to further conversion of PrP^c^ into their pathogenic form [296]. PrP^sc^ form aggregates with itself and other proteins that are highly neurotoxic and lead to severe disruption of normal CNS functions [295]. PrP is commonly found in the diffuse and dense plaques of AD patients and where it can be co-localized with Aβ [297,298,299]. In addition, PrP can directly bind Aβ in both its soluble and membrane-bound form [300,301]. However, the biological consequences of these interactions are still debated because deletion of the PRNP gene did not ameliorate plaque formation in APP/PS1 mice but ameliorated behavioral and cognitive symptoms [302,303]. Interestingly, PrP was shown to modulate BACE1 activity and thus the production of Aβ, but had only diminished effects on processing of the Swedish APP variant [304,305].

## 5. Genetic Variants Affecting Microglial Response to Aβ

Microglia and other glial cells express various receptors and molecules that can either **directly** bind Aβ at the cell surface, which leads to altered cell signaling, or interact with it **indirectly**, which modifies Aβ detection or degradation (Figure 5).

**TREM2** is a cell-surface receptor that is highly expressed in microglia. It mostly detects phospholipids and glycolipids that are released from host-derived apoptotic cells or from invading pathogens (Yeh 2017), but is also able to interact with multiple peptides and proteins such as Aβ and various Apolipoproteins (Apo) including APOE and APOA1 [306]. TREM2 is capable of inducing cell activation and migration towards Aβ_1-42_ [307]. In TREM2-deficient AD mouse models such as modified APP/PS1 and 5xFAD, microglia do not congregate around senile plaques and thus do not respond to the progressing plaque load [306]. In recent years, this receptor has received much attention, since loss of TREM2 leads to nearly complete absence of microglial reactivity in mice [308,309]. In addition, several genome-wide association studies (GWASs) in humans found mutations and polymorphisms of the *TREM2* gene to be a high-risk factor for familiar forms of AD (see Table 2).

**TLRs** comprise a family of pattern recognition receptors (PRRs) that detect pathogen-associated molecular patterns (PAMPs) from exogenous sources such as bacteria and fungi, but can also recognize damage-associated molecular patterns (DAMPs) from endogenous sources [355]. There are ten receptor subtypes (TLR1–10) in humans that are all expressed in the central nervous system (CNS) as well as in other tissues [355,356]. In microglia, TLRs induce cell activation after detection of harmful stimuli and initiate the inflammatory cascade which includes the upregulation of additional PRRs and the production of cytokines and reactive oxygen species (ROS) [357]. In the context of AD, TLR2 and TLR4 are of special interest since they are capable to detect Aβ_1-42_. Both TLR2 and TLR4 mediate the phagocytic uptake and degradation of Aβ in microglia [236,358] and induce typical pro-inflammatory cascades such as the release of Il1β and TNFα as a response to the peptides [359]. Their expression is upregulated in inflamed brain tissue and in plaque-invading microglia of human AD patients [359] but also in microglia derived from APP/PS1 mice [238]. APP/PS1 mice with dysfunctional TLR4 display decreased cytokine levels [360] but also more severe plaque formation [238]. Similarly, TLR2-deficient APP/PS1 animals show accelerated cognitive impairment and increased Aβ_1-42_ concentrations in the brain [237]. On the contrary, in a study with APP/PS1 mice, long-term administration of TLR2-inhibiting antibodies decreased microglial activity, improved cognitive performance, and lowered Aβ plaque load [361].

**CD36** is a class B scavenger receptor that is expressed in microglia, astrocytes, and neurons in the CNS, and in various peripheral cell types such as innate immune cells, myocytes, and endothelial cells [362]. CD36 is mostly known as a fatty acid transporter but is also involved in lipid metabolism and the regulation of inflammatory responses in immune cells [363]. Its ligands include various structural proteins of the extracellular matrix such as collagens and thrombospondins. In addition, CD36 interacts with fibrillary Aβ_,_ which induces a pro-inflammatory activation of microglia [235,364]. Upon detection of Aβ, CD36 mediates cell migration and subsequent binding to Aβ fibrils [364]. It contributes to cytokine production [251] and generation of oxidative stress [234]. In addition, CD36 may act as a co-receptor for other PRRs that interact with Aβ. For example, activation of CD36 can lead to the formation of complexes with TLR2 and TLR6, thus eliciting internalization and degradation of Aβ_1-42_ [365]. Of note, in human brain samples, expression of CD36 is detected in microglia proximal to Aβ plaques [366]. However, Ricciarelli and colleagues reported that CD36 is also highly expressed in healthy people with senile plaques [366].

**RAGE** is a receptor mainly binding advanced glycation endproducts, which are generated by non-enzymatic glycation and oxidation of proteins and lipids [367,368]. Inside the CNS, the receptor is mainly expressed in neurons and glial cells, but can also be found in many other tissues outside the brain [369,370]. Six isoforms of RAGE have been identified. The most common variant is integrated into the membrane (mRAGE), while the other five soluble isoforms lack the transmembrane region (sRAGE) and are thus either cytosolic or secreted into the extracellular space [371]. Moreover, RAGE can be cleaved by ADAM10 and ADAM17, which are also involved in APP processing [18]. RAGE was first associated with AD due to its ability to bind oligomeric Aβ_1-42_, which triggers pro-inflammatory responses in microglia [46,372]. RAGE is involved in Aβ trafficking and clearance through the blood–brain barrier [373] and is thought to modulate secretase activity in neurons, thereby influencing APP processing and Aβ production [374]. Furthermore, RAGE may act as a co-receptor for FPRs that enhances the uptake of Aβ_1-42_ in microglia [46]. More recently, a possible contribution to AD through interaction with Amylin (also known as islet amyloid polypeptide) was proposed [375]. In microglia of APP/PS1, mice expression of human RAGE exacerbates the production of pro-inflammatory cytokines such as Il1β and TNFα, increases the production of Aβ_1-42_ and Aβ_1-40_, and promotes cognitive decline [376]. In contrast, RAGE-deficient APP/PS1 mice showed a decreased concentration of Aβ_1-42_ and Aβ_1-40_ and improved spatial memory [374].

**FPRs** are a small family of G-protein-coupled receptors comprising three receptor subtypes (FPR1, FPR2, and FPR3) in humans [377,378] that are expressed in the innate immune system and a number other cell types, including microglia [379]. FPRs mainly detect formyl methionine-containing peptides that occur in bacteria as PAMPs released during bacterial infections and in mitochondria [380,381], where they are released as DAMPs by damaged cells [381]. They also bind some neuropathological peptides such as Aβ_1-42_ [231,266] and prion protein fragment PrP_106-126_ [382]. In particular, FPR2 has been implicated in AD through its interactions with Aβ_1-42_. In microglia, FPR2 mediates uptake of Aβ_1-42_ [227,229,383] and triggers the inflammatory response against it. In inflamed brain areas of AD patients, FPR2 is upregulated in reactive glia [231]. Interestingly, microglia from APP/PS1 mice show an increase in the murine FPR1 and FPR2 [46], and treatment of APP/PS1 mice with a competitive FPR inhibitor ameliorated cognitive impairment, reduced plaque load, and lessened microglial reactivity [384]. The role of the FPR subtypes in the response to non-canonical Aβ species is not well examined. However, our recent study provided the first in vitro evidence that FPRs can detect N-terminally abridged Aβ species, and that FPR1 and FPR3 may also be involved in detection of Aβ_1-42_ [113].

**MARCO** is a Class A scavenger receptor that is mainly expressed in macrophages and glial cells. It mainly binds poly-anionic ligands such as low-density lipoproteins and environmental particles such as TiO_2_ and Fe_2_O_3_ [385], but has also been shown to capture bacterial PAMPS such as lipopolysaccharides and oxidized lipoproteins, and even helps to engulf whole bacteria in macrophages [386,387,388]. Several studies suggest that MARCO may also bind both fibrillary and non-fibrillary Aβ, either directly or through interactions with other receptors [322]. MARCO modulates intracellular activation of NLRP3 and limits extracellular detection by in glial cells [389]. Furthermore, MARCO can form complexes with FPR2 that facilitate uptake of Aβ_1-42_ and may influence FPR signaling [319].

Chemokine-like receptor 1 (**CMKLR1**) is a G-protein-coupled receptor that is mainly expressed in white adipose tissue and immune cells such as microglia, macrophages, and dendritic cells [390]. CMKLR1 interacts with endogenous ligands such as the adipokine Chemerin, and helps to modulate metabolic processes and cell proliferation, especially during glucose processing, adipogenesis, and angiogenesis [390]. In immune cells, CMKLR1 induces chemotaxis and pro-inflammatory cascades, but is also involved in anti-inflammatory signaling through interactions with pro-resolving ligands such as Resolvin E1 [391]. In brain tissue of AD patients, CMKLR1 is upregulated and co-localizes with Aβ_1-42_ [318]. Furthermore, Aβ_1-42_ binds to the receptor in an in vitro expression system [318]. In primary mouse microglia and cell lines, interactions between CMKLR1 and Aβ_1-42_ induce cell migration, internalization of Aβ_1-42_, and MAPK-dependent inflammatory activation [318]. Knockout of CMKLR1 in APP/PS1 mice leads to increased Aβ deposition but also mortality and cognitive impairment [392]. CMKLR1-deficient mice and wild type mice that were treated with a CMKLR1 inhibitor were more resistant against a chemically induction of Tau hyperphosphorylation [392].

**Nucleolin** is a phosphoprotein mainly distributed in the nucleolus of many cell types, and is also expressed at the cell surface of macrophages and microglia [330,393]. It can bind DNA, RNA, and amyloid-like proteins [393,394]. Inside the nucleus it has been implicated in regulating DNA and RNA metabolism, chromatin structure, and ribosome assembly [394]. At the cell surface it has been implicated in the cellular-entry of various viruses such as human immunodeficiency virus (HIV) and respiratory syncytial virus (RSV). In primary rat microglia, Nucleolin also robustly recognizes monomeric and fibrillary Aβ_1-42_ and mediates its phagocytosis, but shows only weak binding to Aβ_1-40_ [330]. Furthermore, Nucleolin interacts with APP mRNA [395], which may result in modulation and increase APP and Aβ production [396,397].

**TRPM2** is a non-selective calcium-permeable cation channel [398]. Activation of TRPM2 is thought to contribute to warmth-sensing in neurons [399] and to the regulation of cytokine secretion in immune cells [400]. The ion channel does not directly interact with classical ligands but instead senses mediators of oxidative stress such as ROS and excessive nitric oxide [401]. In microglia, detection of Aβ_1-42_ by other receptors leads to the induction of oxidative stress, which in turn activates TRPM2 and induces rapid calcium influx, which triggers cytokine production through the NLRP3 inflammasome [402]. This may lead to a self-renewal of TRPM2 activation since cytokine release can trigger further production of ROS, thus propagating a vicious cycle of pro-inflammatory cascades. Knockout or pharmacological inhibition of TRPM2 in primary mouse microglia attenuates microglial activation by Aβ_1-42_ and inhibits production of TNFα [403]. Next, TRPM2-deficient APP/PS1 mice show improved spatial memory and reduced microglial reactivity, but no change in plaque load [404].

**CD33** is a cell-surface receptor in myeloid cells that is exclusively expressed in microglia within the CNS [405,406]. Through interactions with sialic acids, CD33 can bind glycans and glycolipids. Of note, due to its short extracellular domain, it is assumed that CD33 mainly interacts with ligands on the surface of the cell [406]. In microglia, CD33 does not bind Aβ_1-42_ but is thought to interact with other receptors—especially with TREM2—to modulate their signaling and inflammatory responses [407]. Several GWASs have identified CD33 as a high-risk factor for the development of AD (see Table 2). Expression of CD33 is increased in microglia of AD patients and is positively correlated with insoluble Aβ_1-42_ levels and plaque burden [408]. Deletion of the CD33 gene in microglia derived from human-induced pluripotent stem cells or in primary mouse microglia improves uptake of Aβ_1-42_ [407,408] and leads to increased oxidative burst and production of pro-inflammatory factors [407]. In accordance with these in vitro data, CD33-deficient APP/PS1 mice show reduced brain levels of insoluble Aβ_1-42_ as well as amyloid plaque burden [408]. Injection of a viral system encoding microRNA against CD33 in APP/PS1 mice resulted in reduced expression of CD33 and decreased cerebral levels of soluble Aβ_1-42_ and Aβ_1-40_ [409].

The family of **FcRs** comprises several receptors that are expressed in microglia and many other immune cells, and can bind the Fc-region of different antibody subtypes. Interestingly, plaque-invading microglia show a strong expression of FcRs [410]. In several animal studies, immunization against Aβ_1-42_ led to a significant reduction in plaque load [411,412] and removal of Aβ_1-42_ from CSF [413,414]. A possible explanation is that autoantibodies against Aβ_1-42_ bind aggregates in senile plaques that are then degraded through FcR-mediated clearance by microglia. However, other studies suggest that these effects are independent from FcRs [415]. During the progression of AD, the blood–brain barrier is weakened, which may allow peripheral cells and molecules to enter the brain [411,412]. This permits the entry of antibodies that can bind to Aβ aggregates. A subsequent binding of this complex to FcRs on microglia may help to clear these pathological peptides from affected brain areas [413,414]

Genome-wide association studies (GWASs) and genetic linkage studies associated with late onset AD (reviewed, e.g., in [416,417]) revealed the importance of genes with a regulatory role for microglia and immune responses. In particular, receptors that are expressed on microglia, such as TREM2 and CD33, have been identified by these approaches. This underpins the key role of microglia in perception of Aβ, the regulation of the brain’s inflammatory response to these deleterious peptides, and the removal of the resulting aggregates. For many other putative microglial Aβ receptors (for a summary see Table 2), such as MARCO, identification of AD-related polymorphisms has not yet been carried out. With regard to “binding to Aβ” of these putative receptors, it has to be taken into account that, in most cases, the direct physical binding has not been proven yet. In most cases, binding has been assumed due to pharmacological or genetic interventions in cells or animals. Exemptions are CD14 or TREM2, where immunoprecipitations were conducted [307,340].

Taken together, these observations argue that a delicate balance of differently modified or processed Aβ variants may finally orchestrate immune responses via a number of different microglial receptors that show a differential presence in different microglia subtypes. A detailed analysis regarding Aβ variant-reactivity or potential selectivity of most receptors is missing to date. Moreover, knowledge on the effect of identified polymorphisms of the receptors regarding recognition of modified/abridged Aβ peptides is even scarcer. Another layer of complexity is added by the fact that some mutations may alter the proteolytic processing, either indirectly through microglial receptor signaling or directly through SNPs in the processing proteases that may affect the cleavage pattern, processivity, and shedding. TREM2 SNPs are a good example of such complex mutual effects. They have been shown to accumulate in cell protrusions in close proximity to Aβ plaques [346] that can be cleaved by the metalloproteases ADAM10/17 C-terminal to histidine 157 [17,418,419,420]. The amount of soluble (s) TREM2 is increased in the CSF of AD patients and seems to sustain microglial viability, and to trigger inflammatory responses by the Akt–GSK3β–β-catenin and NF-κB pathway in vitro [421,422]. Consequently, its beneficial capacity lowers ApoE4-related risk of cognitive decline [423]. Carriers of the R47H TREM2 polymorphism show higher levels of sTREM2 in CSF than non-carriers [347]. The p.H157Y variant that was identified in the Han Chinese population within the stalk region of the receptor enhances shedding of TREM2 [419,424]. Other variants, such as the TREM2 p.T66M mutation, led to reduced secretion of a soluble partition of the receptor [347,350]. While the amount of R47H TREM2 on the cell surface remained stable despite elevated secretion of sTREM2 [350], uncleaved plasma-membrane tethered p.H157Y TREM2 was found to be severely reduced [419]. An association of the two SNPs within the human α-secretase ADAM10 promoter, rs514049A/C and rs653765C/T, with AD could not be clearly demonstrated [425,426]. However, the latter was found to be correlated with lowered ADAM10 mRNA levels in PBMCs of CC versus CT/TT carriers and with a lowered level of soluble (s) RAGE in plasma [426]. RAGE is also expressed on microglia [427] and processed by ADAM10 [428]; therefore, the ADAM10 promoter polymorphisms may also play a role in related inflammatory processes in the brain, even if this has not been investigated yet. Moreover, the RAGE polymorphism G82S itself influences shedding as a significant association between G82S genotypes and sRAGE plasma concentrations in samples from non-diabetic/non-obese Koreans [429].

Membrane topology and architecture of the putative or identified Aβ receptors on microglia are quite diverse (see Figure 1), and the intense phagocytic activity of these cells, in general, requires a dynamic membrane architecture. Therefore, polymorphisms in the receptor-encoding genes themselves or in genes of the interaction partners may not only orchestrate Aβ perception. Lipidomic analyses of microglial CD11b-positive small extracellular vesicles from the cryopreserved parietal cortex of a restricted number of patients and controls indicated not only increased levels of TREM2, but also a proinflammatory lipid profile in AD (e.g., increase in the most abundant monohexosylceramide d18:1/24:1 [430]). Moreover, an increase in cholesterol was observed that might influence fluidity of the membrane, in addition to other receptor-modifying pathways such as synthesis of ligands. Sequestration of cholesterol in astrocytes, for example, affected APP processing and accumulation of Aβ peptides [431], and serum starvation induced shedding of BACE1, which could be further aggravated by cholesterol efflux mediated by methyl β cyclodextran [432]. TREM2 itself has been shown to act as a regulator of brain cholesterol metabolism, and TREM2-deficient microglia fail to degrade myelin-derived cholesterol [433]. How its polymorphisms or binding of different Aβ variants might interfere with this function has not been addressed to the best of our knowledge.

In sum, this indicates that activation of microglia by Aβ variants can be affected not only directly by genetic variants of the receptors, but also by their impact on receptor processing or on other functions of the receptors that subsequently reflect on Aβ-driven signaling pathways. A deeper understanding of relevance of the single observed variants for the distinct receptor functions on the microglial surface therefore has to be the focus of research in the future to estimate their potential as therapeutic targets or their relevance in individualized medicine.

## 6. Secondary Structure and Oligomerization Critically Affect Aβ Neurotoxicity

Different aggregate forms of Aβ that lead to plagues have long been suspected to be the major culprit of AD. However, there is clear evidence indicating that the formation of insoluble aggregates alone is insufficient to trigger typical neuroinflammation and neurodegeneration. First, the formation of insoluble Aβ plaques also occurs during normal aging in many people who do not suffer from AD [434,435]. Second, some patients display typical AD symptoms but show only low plaque formation [436]. Third, transgenic APP mice already show a pronounced plaque formation before the onset of neuroinflammatory events [437]. Finally, therapeutic approaches for plaque removal did not ameliorate cognitive symptoms in human patients [9]. This indicates that at least a second factor has to contribute. Increasing evidence suggests that the formation of soluble oligomers and/or differences in the secondary structure might be the second culprit that triggers the initial inflammatory events. For example, soluble Aβ_1-42_ oligomers are highly neurotoxic and can induce strong pro-inflammatory activity in glial cells [438,439]. Next, the Osaka mutation, a familiar form of AD that causes a loss of glutamate at position 22 of Aβ, leads to Aβ peptides with enhanced soluble oligomer formation capability but which are unable to form fibrils, and can therefore not be deposited as insoluble plaques (reviewed in [440]). Studies that injected soluble Aβ_1-42_ oligomers into healthy rodents’ brains observed strong neurodegenerative effects, including aberrant neuroinflammation, synaptic disruption, neuronal death, and cognitive deficits [441,442,443]. For example, Aβ*56, a distinct species of Aβ oligomers with a molecular weight of 56 kDa, was proposed as a promising candidate to explain the genesis of AD, since the injection of such aggregates could induce strong cognitive decline in rat models [444]. However, recent investigations have questioned the validity of these studies [445]. In addition, other studies could not detect Aβ*56 in tissue [439,446] or CSF samples [438,446] of human AD patients or AD model mice. Next, a number of other studies reported no or only mild pathological effects of Aβ oligomer injection into the brain of animals [447,448]. The interpretation of the divergent results is still problematic. However, technical issues cannot be excluded because these studies used a plethora of different protocols that varied strongly in terms of buffer composition, pH value, aggregation kinetics, Aβ concentration, temperature, aggregation time, and agitation. Numerous studies have demonstrated that even small deviations in the environmental conditions lead to drastic differences in secondary structure, the solubility of the resulting aggregates, and their capabilities to elicit biological effects [449,450].

The secondary and tertiary structure of Aβ is an extremely critical factor for its oligomerization and bio-activity (reviewed in [12]). The secondary structure of Aβ_1-40_ and Aβ_1-42_ may assume multiple discrete conformations with α-helix or β-sheet conformers, which can undergo rapid changes depending on environmental factors [451,452]. Aβ_1-42_ possesses two motifs that are capable of forming β-sheets: the highly hydrophobic core motif KLVFFAE (Aβ_16-22_) [453] and the C-terminal region IIGLMVGGVVIA (Aβ_30-41_) [451]. β-sheet formation is essential for aggregation, since β-sheet regions of individual peptides self-assemble into cross-linked structures with parallel or antiparallel organization (cross-β patterns) that self-assemble interlinked fibrils and protofibrils [12,454,455]. This self-assembly from monomers in solution is first auto-catalyzed by the formation of nuclei, small aggregates with especially high thermodynamic energy states. These energy levels lead to faster association than dissociation rates of monomers and small oligomers to the nucleus and thus drive further aggregation [456]. This process is enhanced through secondary nucleation where other fibrils or protofibrils in the solution associate with the initial nucleus and accelerate its growth. The nucleation and growth phase finally ends in fibril formation since the fibrillary state is thermodynamically the most stable form of any peptide assembly [456]. In particular, the inflexible hydrophobic C-terminus of Aβ is thought to initiate the transformation from α-helical to β-sheet structure that is needed for the formation of nuclei [12,451]. Accordingly, C-terminal truncation decreases the hydrophobic region and increases the structural flexibility of Aβ, which in turn decreases its propensity to form β-sheets. This may explain why Aβ species with longer C-termini tend to be more amyloidogenic then shorter variants.

Of note, many soluble Aβ variants and their oligomers are present as metastable species with distinct thermodynamic energy levels [457,458]. Some studies have proposed the existence of a α-pleated sheet conformation in a subset of these soluble Aβ oligomers that might set them apart from non-toxic fibrils and are not generated by typical nucleation [459,460,461]. The α-pleated sheet conformation is thought to be structurally similar to β-sheets but may exhibit a distinct biological effect. However, the biological relevance of α-pleated sheet conformations is still debated. A recent study by Shea and colleagues utilized probes designed to catch α-pleated sheet peptides in a transgenic Aβ *Caenorhabditis elegans* model and in transgenic APP mice, which resulted in a reduction in soluble Aβ oligomers in both models [460].

Taken together, these findings highlight the high importance of keeping track of Aβ’s secondary structure during the investigation of its biological effects. This is especially necessary in studies performed with synthetic Aβ peptides. In general, synthesis processes are not performed under physiological conditions and, therefore, can produce peptides with altered conformations [462]. During synthesis, Aβ peptides already aggregate; thus, there is little control over the Aβ species that comprise the final end product [462]. Next, most peptide synthesis processes depend on counter ions such as trifluoracetate or hydrochloride, which are bound to the final synthesis product and can highly influence the solubility, nucleation, and aggregation kinetic of generated Aβ peptides [463,464]. Thus, the use of Aβ peptides derived from different companies and synthesis processes can produce largely different biological effects [113,465], which may additionally vary from batch to batch [456,465]. Various methods have been developed to minimize these aggregation artefacts, e.g., dissolving synthetic Aβ in harsh solvents such as DMSO, HFIP, or NH_4_OH [450,466,467]. However, several studies provided evidence that these treatments can compromise the secondary structure of Aβ [468,469].

Taken together, these findings highlight the need for standardization protocols that improve the comparability and reproducibility of Aβ research. All in vitro experiments should be performed with Aβ obtained from at least two independent sources to validate the observed biological effects and to avoid potential artifacts [113,456,465]. Inexpensive methods such as Thioflavin T aggregation assays or analysis via SDS-PAGE should be used to give some insight regarding the aggregation state of Aβ. Next, all experimental conditions have to be described very carefully because the exact assay buffer composition, pH value, incubation time, assay temperature, and Aβ storage conditions can all influence the outcome of experiments. Finally, it is essential to also report negative or non-conclusive data obtained with different Aβ variants and manufacturers, since these can still provide valuable information for other researchers and may help to elucidate the bigger picture of the still mysterious Aβ.

## 7. Concluding Remarks

In summary, the currently available data clearly argue for a significant contribution of non-canonical Aβ variants to the pathogenesis and progression of AD. However, there is still an amazingly large lack of knowledge on the precise contribution of most of these variants. Next, it seems that the influence of gene polymorphisms regarding the recognition of modified or abridged Aβ peptides is even scarcer. Amazingly, only 20 polymorphisms of Aβ interaction partners have so far been clearly associated with AD. Given that for TREM2 alone nearly 200 single nucleotide exchanges have been reported, and that at least 16 other interaction partners exist, presumably thousands of additional SNPs still await a careful analysis of their impact on receptor interaction, proteolytic processing, and shedding. Next, there is clear evidence that microglia, astrocytes, and oligodendrocytes are key players in the production and release of non-canonical Aβ variants. Therefore, the interplay of these cell types with neurons has to be better characterized. Finally, there is a clear need for standardized protocols in in vitro studies and more information on the Aβ peptide composition in in vivo studies. The current literature already provides ample evidence that chemical modifications, extracellular environment, and precise quantities of different amyloid species have a strong influence on the aggregation and bio-activity of Aβ variants. In a recent study, we even showed that supposedly identical Aβ peptides are highly subjectable to solvent- and manufacturer-dependent effects [113]. For the sake of reproducibility, it is thus of uttermost importance to work with more than one peptide in all in vitro experiments, to very precisely report all experimental conditions, and to include data on the secondary structure, 3D conformation, and aggregation kinetics [113,456,465]. In vivo studies urgently need to gather more information on the precise Aβ variant composition, the existing oligomers, and their 3D structure. This will help to identify the key variants and their structural requirements for a given physiological effect.

## Figures and Tables

**Table 2:** **Microglial Aβ-binding/binding-modulating receptors and identified AD-associated polymorphisms and mutations.** Search for polymorphisms and mutations was conducted using PubMed text search or UniProt https://www.uniprot.org/uniprotkb/.../entry#disease_variants, accessed from 20 June to 4 August 2022. * In the case that no association with AD has been described yet, known polymorphisms or mutations affecting receptor functions are listed.

Receptor	Alternative Receptor Names	Binding to Aβ Demonstrated in	Ref.	Function	Polymorphisms/ Mutations Associated with AD	Ref.
Amylin receptor	calcitonin receptor and receptor activity modifying protein 3	human fetal microglial (HFM) cultures and BV-2, oligomeric soluble Aβ_1-42_	[310]	Increase IL-6 secretion	No association with AD described Cys40Trp/Phe100Ser/Leu147Pro variant associated with reduced Amylin potency *	[311]
CD33	SIGLEC3	No direct binding	-	Blockade of TREM2	rs3865444C	[312,313,314,315]
CD36	Platelet glycoprotein 4 Fatty acid translocase (FAT) Glycoprotein IIIb (GPIIIB) PAS IV	Primary murine microglia, fibrillary Aβ _1-42_	[235]	Increase ROS production and Aβ phagocytosis	rs7755 rs3211956 rs3211892	[316] [317]
CMKLR1 (Chemokine-like receptor 1)	Chemerin-like receptor 1 1 G-protein-coupled receptor ChemR23 G-protein-coupled receptor DEZ	stably transfected rat basophilic leukemia (RBL) cells, primary microglia, N9 cells, Aβ-_1-42_	[318]	Activation of G proteins and β-arrestin pathways	No association with AD described	-
FPR1/2 (fMet-Leu-Phe receptors 1/2)	N-formyl peptide receptor (FPR) N-formylpeptide chemoattractant receptor	Rat primary astrocytes and microglia,	[319]	Intracellular calcium mobilization, cell migration and superoxide anion release	No association with AD described	-
		human Aβ_1–42_	[113]			
		transfected HEK293 cells and glial U87 cells, Aβ_42_ and N-truncated forms				
MAC1 (Macrophage antigen complex 1)	integrin CD11b/CD18 receptor CR3	Primary microglia enriched culture, mice, Aβ-_42_	[320]	Adhesive interactions mediation of the uptake of complement-coated particles and pathogens	No association with AD described	
						[321]
					Various polymorphisms/mutations in CD18 causing leukocyte adhesion deficiency, e.g., rs552407409 *	
MARCO (Macrophage receptor with collagenous structure)	SCARA2	Microglia from neonatal rats, fibrillary and non-fibrillary Aβ-_42_	[319,322]	Inflammatory response	No association with AD described	-
NMDA-R (NMDA receptor)		glial cells in rat cerebellar granule cell cultures, small Aβ_1-42_ oligomers	[323]	Decrease in plasma membrane potential	-421C/A in sporadic AD, North Han Chinese populations	[324] [325]
					3723 G/A (rs3739722), Taiwanese population	[326]
					rs1806201 T, Southern Italy population	[327]
					rs10845840, US populations	[328]
					C2664T, Chinese Han population	not found in [329]
Nucleolin	Protein C23	EOC2 cells, transfected HEK cells, monomeric and fibrillary Aβ_1-42_	[330]	Chromatin decondensation, pre-rRNA transcription, and ribosome assembly	No association with AD described	-
RAGE (Receptor for advanced glycosylation end products)	-	Human primary microglia, soluble Aβ and plaques	[331]	instigates pro-inflammatory mediators	G82S	[332,333,334]
cAI (Scavenger receptor type AI)	Macrophage scavenger receptor types I and II Macrophage acetylated LDL receptor I and II CD204	human fetal microglia, microglia from newborn mice, fibrillary aβ	[233]	Phagocytosis of soluble and fibrillar Aβ	No association with AD described	-
SRBI (Scavenger receptor class B member 1)	CD36 and LIMPII analogous 1 (CLA-1) CD36 antigen-like 1 Collagen type I receptor, thrombospondin receptor-like 1	human fetal microglia, microglia from newborn mice, fibrillary aβ	[233]	Decreases amyloid fibrillar and plaque formation	Gene is included in a region on chromosome 12 with linkage to AD [335], polymorphisms were not found associated	[336]
					rs387906791, rs74830677, impact on cholesterol metabolism *	[337] [338]
SRCL (scavenger receptor with C-type lectin)	Collectin-12 Collectin placenta protein 1 (CL-P1; hCL-P1) Nurse cell scavenger receptor 2 Scavenger receptor class A member 4	CHO-K1 cells, fibrillary A-β	[339]	Aβ and Gram-positive, Gram-negative bacteria and yeast phagocytosis	No association with AD described	-
TLR2/ TLR4/ CD14 (Toll-like receptor 2/4/6/ CD14)	CD282/CD284/ Monocyte differentiation antigen CD14	CD14: soluble murine, fibrillary human Aβ-_42_	[340]	Instigates inflammatory response and Aβ phagocytosis	P249S (TLR6) 260C/T (CD14)	[341] [342]
		CD14/TLR2/4: BV2 microglia, fibrillary Aβ	[343]			
TREM1 (Triggering receptor expressed on myeloid cells 1)	CD354	mouse primary microglia, monomeric Aβ_1-42_	[65]	Amplifying inflammatory responses	rs6910730G	[344]
TREM2 (Triggering receptor expressed on myeloid cells 2)		immobilized TREM2-FC, oligomeric and monomeric Aβ_1-42_	[307] [345]	Regulates microglial activity, chemotaxis, and process outgrowth	R47H R62H	[346,347,348,349] [350,351] [352] [353]
					H157Y D87N	
α6β1 integrin	CD49 antigen-like family member F VLA-6 CD49f/ Fibronectin receptor subunit β Glycoprotein IIa (GPIIA) VLA-4 subunit β CD29	BV-2 cells, fibrillary Aβ_25-35_ and Aβ_1-42_	[354]	increase ROS production and Aβ phagocytosis	No association with AD described	

## Data Availability

Not applicable.
